# Patient Blood Management in Brazil: between evidence, reality and transformation

**DOI:** 10.1016/j.bjane.2026.844738

**Published:** 2026-02-28

**Authors:** Guilherme Machado Rabello, Angel Augusto Pérez-Calatayud, Luís Vicente Garcia, Liana Maria Tôrres de Araújo Azi

**Affiliations:** a Escola Politécnica da Universidade de São Paulo (USP), São Paulo, SP, Brazil; b InovaInCor, Instituto do Coração, Hospital das Clínicas, Faculdade de Medicina, Universidade de São Paulo (USP), São Paulo, SP, Brazil.; c Sociedad Iberoamericana de Patient Blood Management (SIAPBM), Mexico; d Board of Directors, Society for Advancement of Patient Blood Management (SABM), USA; e Critical Care Division Hospital General de México Dr. Eduardo Liceaga, Mexico.; f Faculdade de Medicina de Ribeirão Preto da Universidade de São Paulo (USP), Ribeirão Preto, SP, Brazil; g Universidade Federal da Bahia (UFBA), Salvador, Brazil.

Nearly two decades after the formal consolidation of the Patient Blood Management (PBM) concept,^1^ the global healthcare community now benefits from a substantial and consistent body of evidence demonstrating its association with improved clinical outcomes, rational utilization of blood components, and enhanced sustainability of healthcare systems.^2^ PBM has evolved beyond a set of procedural steps into a comprehensive strategy that prioritizes patient-centered, physiology-based care, seeking to minimize unnecessary allogeneic transfusion, optimize the management of anemia and bleeding, and foster evidence-based clinical decision-making.^3^ This evolution reflects not only advances in clinical science but also a growing recognition that blood is a finite and precious healthcare resource with significant implications for patient safety, system costs, and quality of care.

Despite this robust evidence and clear clinical rationale, the practical implementation of PBM remains heterogeneous across healthcare settings worldwide – particularly in environments marked by structural complexity, inequity, and operational constraints.^4^ These conditions are exemplified by the Brazilian healthcare context, where disparities in infrastructure, workforce distribution, and data systems intersect with broader socioeconomic challenges. In Brazil's large and diverse health system – one of the world's most expansive public healthcare systems – translating PBM from concept into practice has revealed not only clinical barriers but also systemic ones that reflect broader health system strengths and limitations.^5^

PBM was initially conceived as a technical framework structured around three foundational pillars: optimization of erythropoiesis through the identification and treatment of anemia, minimization of blood loss through evidence-based surgical and anesthetic practices, and enhancement of physiologic tolerance to anemia when appropriate.^1^^,^^6^ However, its evolution has revealed a broader scope. PBM is now understood as a model of care reorganization that requires cultural transformation, coordinated clinical governance, multidisciplinary integration, and institutional alignment. This inherently transformative character explains, in part, the persistent gap between scientific evidence and routine clinical practice.^7^

Even with compelling evidence supporting improved patient outcomes and reduced inappropriate blood use, adoption of PBM remains uneven. International experience indicates that successful PBM adoption is contingent upon factors extending beyond dissemination of clinical protocols.^8^ Jurisdictions that have demonstrated sustained progress have combined strong clinical leadership – often in the form of PBM "champions" – with institutional engagement, structured data governance, and alignment with national health policy frameworks. These factors create an enabling environment in which PBM practices can be standardized, monitored, and continuously refined.^9^

The Brazilian healthcare landscape illustrates these tensions with notable clarity. Brazil operates one of the world's largest public health systems, the Sistema Único de Saúde (SUS), which serves more than 200 million individuals and coexists with a technologically sophisticated private sector. At the same time, the system is marked by pronounced regional disparities, fragmentation of care delivery, and variability in infrastructure capacity. These disparities influence not only access to care but also capacity for consistent implementation of complex care models like PBM.^10^

Brazil's blood donation and transfusion ecosystem provides an instructive example. Recent official data indicate that approximately 1.9% of the Brazilian population donated blood in 2024, accounting for roughly 3.3 million units collected nationally. This rate is within the World Health Organization's recommended range of 1–3% blood donors per population, but it remains low compared with many high-income countries and reflects persistent challenges in building a stable donor base. Despite this modest donor rate, blood usage in Brazil remains high. In 2024, over 3.1 million blood component units were transfused in SUS facilities, reflecting both the scale of demand and the complexity of clinical care delivered.^11^ This discrepancy between collection and utilization highlights a critical challenge: managing scarce blood resources efficiently while meeting the needs of patients requiring transfusions for trauma, surgery, cancer care, hematologic conditions, and obstetric emergencies. In many cases, such demand occurs within systems that lack advanced preoperative anemia management programs or standardized PBM pathways, amplifying reliance on transfusion rather than evidence-based alternatives.

Recent Brazilian data underscore the magnitude of this challenge. A retrospective cohort study conducted in a university hospital in northeastern Brazil evaluated 508 patients undergoing major elective surgeries and found that preoperative anemia was present in 59.6% of patients, with mean hemoglobin levels of 11.66 g/dL in women and 11.13 g/dL in men. More strikingly, postoperative anemia increased to 94.6%. Patients with preoperative anemia were 4.6 times more likely to require intraoperative transfusion compared to non-anemic patients (OR = 4.58; 95% CI: 2.78−7.52; p < 0.001). These findings highlight the critical importance of preoperative anemia detection and management as a cornerstone of PBM implementation in Brazilian surgical practice.^12^

The association between preoperative anemia and adverse perioperative outcomes has been further analyzed by a large retrospective cohort study involving 23,579 adults undergoing elective noncardiac surgery. This study demonstrated that preoperative anemia, even when mild (hemoglobin 11.1 to 12.9 g/dL), was independently associated with higher in-hospital mortality, a greater ICU admission rate, and a prolonged hospitalization. Severe anemia (hemoglobin < 8.0 g/dL) emerged as the strongest predictor of in-hospital mortality (adjusted RR 24.7; 95% CI 13.3-46.0; p < 0.001). Notably, these adverse associations persisted even in patients that were ASA I and II, undergoing low-risk procedures, challenging a therapeutic inertia that often leads clinicians to overlook anemia in seemingly "simple" surgical scenarios.^13^

Regional variations further complicate the picture. In the state of São Paulo alone, government data reported 789,000 units of blood collected in 2024, up from 721,000 the previous year – a 9.5% increase – reflecting localized improvements in donation campaigns and infrastructure.^11^^,^^14^ Meanwhile, states such as Ceará recorded 110,784 units collected in 2024, the highest in decades, indicating areas of excellence that nevertheless remain isolated.^15^ These localized increases are encouraging, yet they underscore the heterogeneity of blood donation systems across Brazil's regions and the need for national coordination to scale effective practices.

The interplay between blood collection rates and broader health system indicators also reveals deeper structural considerations. Brazil's health system must respond to a broad spectrum of healthcare needs, from infectious and chronic diseases to emergency care and advanced surgical interventions. In 2024, the SUS coordinated over 30,000 organ transplants, an 18% increase compared with 2022, demonstrating the complexity of care delivered in the public system. Such high complexity care often relies on safe, timely transfusion support but also highlights the importance of integrated care pathways that can reduce unnecessary interventions and optimize resource use.^16^

The persistent reliance on transfusions reflects, in part, entrenched clinical paradigms. Transfusion medicine has historically been viewed as an immediate and inherently benign intervention, and this perception persists among many clinicians.^17^ Contemporary evidence, however, underscores both the clinical risks associated with allogeneic blood transfusion – including immunomodulation, infection risk, and increased length of stay – and the potential for PBM to improve outcomes by reducing unnecessary exposure to blood products.^18^ This shift in evidence has spurred international calls for PBM adoption as a standard of care, yet changing deeply rooted clinical norms requires sustained educational efforts, interprofessional collaboration, and institutional reinforcement.

Beyond the traditional transfusion management, PBM implementation in surgical scenarios requires perioperative monitoring of coagulation and hemostasis. A recent randomized clinical trial conducted in Brazil evaluated the accuracy of point-of-care CoaguChek XS testing versus standard laboratory coagulation monitoring in 50 patients undergoing cardiac surgery with cardiopulmonary bypass. The study demonstrated that CoaguChek XS provided results comparable to standard laboratory methods both pre- and post-cardiopulmonary bypass, with Lin's concordance correlation coefficients of 0.72 (95% CI: 0.60–0.82) pre-CPB and 0.66 (95% CI: 0.50–0.77) post-CPB, both indicating good agreement. Although statistically significant differences were observed, they fell within the predefined clinically irrelevant tolerance range of ± 0.5 INR units. This validation of point-of-care testing in the Brazilian context is particularly important, as it provides evidence for the adoption of rapid coagulation monitoring strategies that can facilitate real-time clinical decision-making in perioperative settings, especially in resource-constrained environments where laboratory turnaround times may be prolonged.^19^

Cultural resistance remains a primary barrier in many Brazilian institutions. Variability in professional training, unequal access to advanced diagnostics (such as accurate anemia work-up tools), and high care demand environments contribute to hesitation in adopting PBM practices. Clinicians often lack familiarity with structured anemia pathways or may feel constrained by time pressures and established workflows that do not prioritize preoperative optimization. These human and organizational factors are as significant as technical ones and underscore the need for comprehensive educational strategies integrated into medical, nursing, and allied health curricula.^18^

Structural limitations constitute another critical challenge. Effective PBM implementation requires organized pathways for anemia screening and management, systematic clinical risk assessment, availability of alternatives such as intravenous iron and erythropoiesis – stimulating agents, coordinated perioperative planning, and interdisciplinary care teams. In many healthcare settings across Brazil, deficiencies in health information systems impede the generation of actionable data required to identify quality gaps, monitor performance, and support continuous improvement. In the absence of reliable metrics, PBM risks being perceived as an optional or aspirational initiative rather than a core institutional strategy.^4^

Health information infrastructure is central to overcoming this barrier. Robust systems such as DATASUS – Brazil's primary national health data repository – exist to collect and process health indicators across epidemiologic, clinical, and administrative domains.^16^ However, the utility of such systems for PBM implementation remains underleveraged. Integration of transfusion data, preoperative anemia prevalence, and patient outcomes into interoperable dashboards could facilitate performance tracking and stimulate continuous improvement at local and national levels.

The broader contextual environment also influences implementation. Healthcare systems under financial constraints frequently prioritize immediate therapeutic interventions over preventive strategies, particularly in environments where acute care demand is high. Paradoxically, although PBM has been consistently associated with cost containment and efficiency gains – including reduced blood unit expenditure, shorter hospital lengths of stay, and decreased transfusion-related complications – it requires upfront investments in infrastructure, training, and process reorganization.^2^^,^^20^^,^^21^ This temporal disconnect between investment and measurable return represents a tangible barrier to administrative adoption, especially when short-term budget pressures dominate decision-making.

Despite these challenges, meaningful progress is evident within Brazilian clinical and professional communities. Leading medical associations, including the Brazilian Society of Anesthesiology (SBA) and the Brazilian Association of Hematology, Hemotherapy and Cellular Therapy (ABHH), have increasingly incorporated PBM principles into clinical guidelines, educational activities, and consensus documents.^18^ These efforts signal recognition of PBM as not only a clinical innovation but also a strategy aligned with quality improvement and long-term sustainability objectives.

At the institutional level, both public and private hospitals have initiated structured PBM programs with promising clinical and operational outcomes. These include preoperative anemia screening protocols, enhanced hemostasis strategies, and blood utilization review committees that provide real-time feedback to clinicians. Early data from these initiatives suggest improved perioperative management and reduced transfusion rates, mirroring international PBM experience.^22^^,^^23^

Clinical champions continue to play a pivotal role in catalyzing PBM adoption. National and international experience demonstrates that many successful programs originate from professionals capable of integrating scientific evidence, educational initiatives, and management strategies.^9^^,^^10^ However, enduring sustainability requires institutionalization. PBM must transcend individual advocacy and be embedded within formal governance structures supported by measurable objectives, performance indicators, and executive endorsement. Institutional leadership involvement – including hospital directors and clinical chiefs – is essential to formalizing PBM within organizational priorities and resource allocation frameworks.

Data governance represents another essential dimension. PBM is intrinsically metrics-driven. Monitoring indicators such as preoperative anemia prevalence, transfusion rates, complication profiles, and cost parameters enables continuous evaluation and refinement of clinical strategies. In a country of continental scale, collaborative networks that facilitate shared learning and benchmarking across institutions could significantly accelerate performance improvement and reduce regional disparities. Standardized data collection and transparent reporting are essential to creating this learning environment.^20^

Within the global context, Brazil shares characteristics with other middle-income healthcare systems – including Mexico, Colombia, India, and parts of Africa – where PBM implementation encounters comparable infrastructure and governance challenges. Yet Brazil also possesses distinctive strengths: demographic scale, a universal public health framework, expanding technological capacity, and a growing community of PBM advocates within clinical sciences.^18^ These attributes create a unique opportunity to develop hybrid implementation models that integrate clinical innovation, health policy alignment, and organizational governance.

Advancing PBM implementation requires a structured strategic roadmap. Priority actions should include: embedding multidisciplinary PBM education within professional training programs; formal institutional recognition of PBM as a quality-of-care initiative; strengthening of health information systems to enable real-time performance tracking; alignment with national and regional health policies that recognize PBM as a sustainability strategy; and development of collaborative networks to facilitate knowledge exchange, benchmarking, and progressive standardization.^3^

Establishing robust performance indicators is vital for translating PBM principles into measurable outcomes and to ensure accountability at institutional and system levels. Key performance indicators (KPIs) for PBM implementation should encompass both the process measures and the outcome measures, enabling continuous monitoring and quality improvement.^24^ In Brazil's context, where health information systems such as DATASUS exist, but remain underutilized for PBM monitoring, establishing standardized KPI dashboards could really facilitate real-time performance tracking across SUS facilities and private hospitals. A systematic approach to KPI selection and measurement has been demonstrated to improve program effectiveness and sustainability.^25^

Beyond a collection of clinical protocols, PBM embodies a paradigm of patient-centered, physiology-based, and evidence-informed care. Its implementation serves as a marker of a healthcare system's capacity to translate scientific knowledge into operational practice. In Brazil, this transformation is ongoing – complex, uneven, but directionally irreversible. Successful PBM implementation requires establishing systematic monitoring mechanisms and feedback loops that can engage clinical teams in continuous improvement cycles. Data collection should be conducted prospectively using standardized definitions and electronic health record systems to ensure consistency and reduce measurement bias.^26^ In resource-constrained Brazilian settings, establishing collaborative networks among institutions can facilitate shared learning and benchmarking, allowing the hospitals to compare their performance against regional and national standards. The ultimate impact of PBM extends beyond traditional transfusion metrics. It reflects the institutional maturity required to integrate culture, governance, and clinical management in pursuit of safer, more efficient care delivery. In healthcare systems of substantial complexity, such transformation is challenging but indispensable.^3^

The Iberoamerican Society of Patient Blood Management (SIAPBM) was established with the mission of advancing PBM as a standard of quality, safety, and sustainability across Ibero-American healthcare systems. Its vision aligns with global recommendations from the World Health Organization and the International Foundation of Patient Blood Management (IFPBM), while recognizing the structural and socioeconomic particularities of low and middle-income countries. SIAPBM conceptualizes PBM not merely as a clinical innovation, but as a regional health policy movement designed to standardize PBM principles across Ibero-American healthcare systems**,** strengthen governance and institutionalization of PBM**,** develop certified PBM implementers and multidisciplinary leadership**,** promote data-driven implementation models**,** foster collaborative regional research networks and align PBM with sustainability and economic efficiency frameworks.^27^

Within Brazil’s complex health ecosystem, characterized by the coexistence of the Sistema Único de Saúde (SUS) and a technologically advanced private sector, SIAPBM’s strategic roadmap provides a structured pathway to bridge the persistent gap between scientific evidence and operational practice. The Society’s regional perspective positions Brazil not only as a beneficiary of PBM implementation, but as a continental leader capable of developing scalable hybrid models that integrate: Clinical excellence, data governance modernization, policy alignment and multidisciplinary cultural transformation. In this framework, PBM becomes a measurable quality indicator of institutional maturity. The integration of SIAPBM objectives into Brazil’s implementation strategy reinforces the idea that PBM is not a discretionary innovation, but a structural reform aligned with patient safety, economic sustainability, and ethical stewardship of blood as a finite public resource.^28^ This vision recognizes that sustainable PBM adoption requires, institutional embedding within hospital governance, formal KPI monitoring linked to executive accountability, continuous professional education integrated into residency and specialist training, regional benchmarking networks to reduce inequities and policy level recognition of PBM as a national health priority that represents more than clinical optimization, it reflects the healthcare system’s capacity to operationalize science, integrate governance, and transform professional culture toward safer, physiology based, patient-centered care. In this evolving landscape, SIAPBM functions as a regional accelerator of structured implementation, ensuring that PBM progresses from isolated institutional initiatives to a coordinated, measurable, and policy-aligned continental strategy.^27^

PBM should therefore be understood not merely as a clinical strategy but as a test of a system's ability to harmonize scientific evidence, organizational governance, and professional culture in service of sustainable, high-quality patient care.

## Declaration of competing interest

The authors declare no conflicts of interest.
